# Survival Benefit of Experience of Liver Resection for Advanced Recurrent Hepatocellular Carcinoma Treated with Sorafenib: A Propensity Score Matching Analysis

**DOI:** 10.3390/curroncol30030243

**Published:** 2023-03-09

**Authors:** Kuan-Chun Hsueh, Cheng-Chun Lee, Pi-Teh Huang, Chih-Yu Liang, Shun-Fa Yang

**Affiliations:** 1 Division of General Surgery, Department of Surgery, Tungs’ Taichung Metroharbor Hospital, Taichung 435, Taiwan; 2 Department of Post-Baccalaureate Medicine, College of Medicine, National Chung Hsing University, Taichung 402, Taiwan; 3 Division of Diagnostic Radiology, Department of Medical Imaging, Tungs’ Taichung Metroharbor Hospital, Taichung 435, Taiwan; 4 Division of Gastroenterology and Hepatology, Department of Internal Medicine, Tungs’ Taichung Metroharbor Hospital, Taichung 435, Taiwan; 5 Department of Nursing, Tungs’ Taichung Metroharbor Hospital, Taichung 435, Taiwan; 6 Institute of Medicine, Chung Shan Medical University, Taichung 402, Taiwan; 7 Department of Medical Research, Chung Shan Medical University Hospital, Taichung 402, Taiwan

**Keywords:** advanced hepatocellular carcinoma, surgical resection, postoperative recurrence, sorafenib, survival outcome

## Abstract

Several studies have shown that liver resection (LR) confers better survival outcomes in intermediate- and advanced-stage hepatocellular carcinoma (HCC) patients. However, the postoperative recurrence rate is high, and little is known about the survival benefits of LR for recurrent HCC patients who have already received systemic treatment. This study aimed to evaluate the impact of LR on recurrent advanced-stage HCC patients who received sorafenib as a systemic treatment. In this study, 147 advanced HCC patients were enrolled between 1 January 2012 and 31 December 2019. Two study groups were classified, based on whether they underwent LR or not. To reduce the possible selection bias, a propensity score matching (PSM) analysis was performed. The primary study endpoint was set as overall survival (OS), and the secondary endpoint was set as progression-free survival (PFS). Our study results revealed that advanced HCC patients who received sorafenib with LR had a longer OS than did those without LR, whether before or after PSM (15.0 months vs. 6.0 months, HR 0.45, 95% CI 0.31–0.67, *p* < 0.001; 15.0 months vs. 5.0 months, HR 0.46, 95% CI 0.28–0.76, *p* = 0.001). Similar results were obtained in PFS, before or after PSM (4.14 months vs. 2.60 months, HR 0.60, 95% CI 0.40–0.89, *p* = 0.01; 4.57 months vs. 2.63 months, HR 0.58, 95% CI 0.34–0.97, *p* = 0.037). Multivariate analysis showed that the experience of LR was independent of other factors associated with better OS and PFS, whether before or after PSM (*p* < 0.05). Therefore, advanced HCC patients who have undergone liver resection should be encouraged to continue sorafenib treatment to improve prognosis.

## 1. Introduction

Hepatocellular carcinoma (HCC) is the fourth most common cause of cancer-related deaths worldwide [1]. HCC is usually diagnosed at an advanced stage, with a poor prognosis and an even lower possibility of receiving curative treatment [2,3]. The most widely used Barcelona Clinic Liver Cancer (BCLC) system recommends liver resection (LR) for patients with very-early-stage and early-stage HCC, while transcatheter arterial chemoembolization (TACE) and sorafenib therapy are recommended for those with intermediate-stage and advanced-stage HCC, respectively [4,5,6]. However, this clinical practice guideline is not always appropriate for every patient, due to the heterogeneity of HCC, exampled of which include tumor size, number of tumors, vascular invasion, and the patient’s liver function [7]. Increasingly, studies have reported better survival outcomes in selected stage B or C patients who undergo LR, compared to those receiving non-surgical treatment [8,9,10,11]. Unfortunately, the likelihood of HCC recurrence after LR is up to 60–70% within 5 years [12]. Therefore, there are ongoing concerns regarding the treatment and prevention of this high postoperative tumor recurrence, and the amelioration of liver function impairment due to recurrence in advanced HCC patients. Sorafenib, a multi-targeted tyrosine kinase inhibitor (TKI) with anti-angiogenic and anti-proliferative properties, has been found to prolong overall survival in advanced HCC patients in two phase III studies [13,14]. Little is known about the survival benefit of sorafenib for advanced HCC patients, with or without the previous experience of LR.

Therefore, we enrolled BCLC stage C patients who were classified as Child-Turcotte-Pugh (CTP) class A and treated by sorafenib as systemic therapy in this study. The cohort was divided into two groups: those with and those without previous experience of LR. The clinical outcomes between these two groups were compared using real-world data with propensity score matching (PSM). In addition, prognostic factors of survival were investigated.

## 2. Materials and Methods

### 2.1. Patients and Study Design

**Figure 1:**
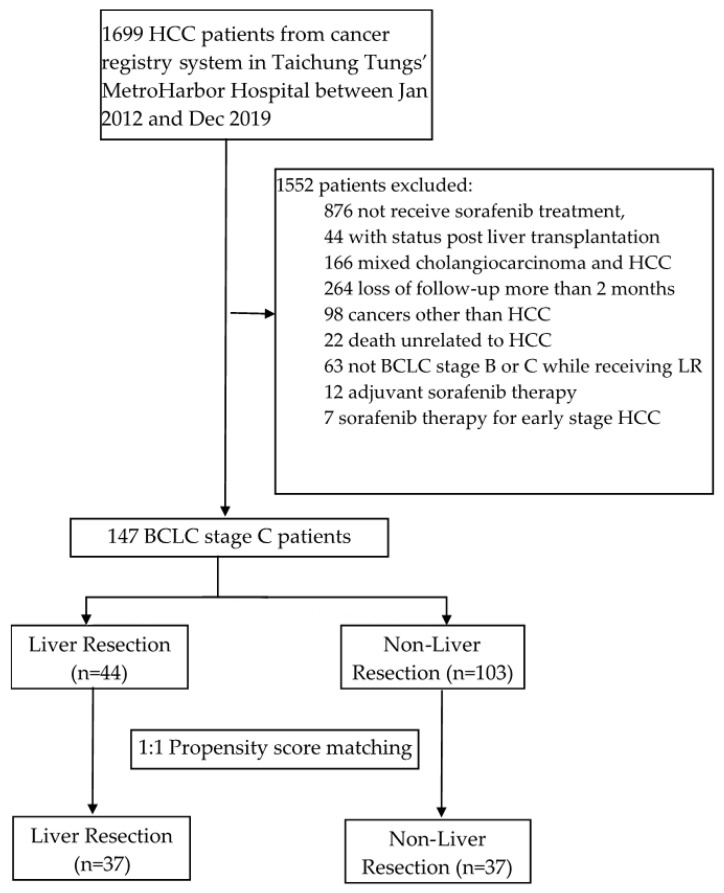
Flow chart of the study.

A total of 1699 HCC patients were retrospectively screened from the cancer registry system at a regional hospital (Tungs’ Taichung MetroHarbor Hospital) between 1 January 2012, and 31 December 2019 (Figure 1). Patients who had been diagnosed with HCC using magnetic resonance imaging (MRI) or contrast-enhanced computed tomography (CECT), from the diagnostic criteria of the American Association for the Study of Liver Diseases (AASLD) clinical practice guidelines [15], and been classified as advanced-staged (BCLC stage C), were enrolled in the study. Patients were excluded if they did not receive sorafenib treatment, received sorafenib with the diagnosis of early- stage HCC, had undergone liver transplantation, had mixed cholangiocarcinoma and HCC following surgical pathology, were lost to follow-up for more than 2 months, had cancers other than HCC, had died for reasons unrelated to HCC, or were not diagnosed as BCLC stage B or C while receiving surgical resection. A total of 159 patients were initially selected for this research; however, 12 HCC patients using sorafenib as adjuvant therapy were excluded. A total of 147 patients were included and were then divided into two groups: patients with or without previous experience of LR (Figure 1).

The patients’ demographic and biochemistry data within 2 months before the use of sorafenib were recorded. These data incorporated age, gender, the etiology of hepatitis, serum albumin level, serum total bilirubin level, alpha-fetoprotein (AFP) level, Eastern Cooperative Oncology Group (ECOG) performance status, the presence of ascites, and CPT score. The tumor burden, which is represented as the combined presence of extrahepatic spread (EHS), portal venous thrombosis (PVT), larger size (>3 cm), and multi-nodularity (equal to or more than 3 in number), was also recorded.

### 2.2. Outcomes and Evaluation

The treatment response was evaluated according to the Response Evaluation Criteria in Solid Tumors version 1.1 (RECIST v1.1) every 2–3 months. The primary endpoint was overall survival (OS), and the secondary point was progression-free survival (PFS). The cut-off date for both OS and PFS analyses was 31 December 2020. The OS was defined as the time period between the start date of use of sorafenib and death due to HCC. The PFS was defined as the time period between the start of the sorafenib treatment and disease progression.

### 2.3. Ethical Considerations

This study was approved by the ethical committee of Tungs’ Taichung MetroHarbor Hospital and performed according to the principles of the 1975 Declaration of Helsinki.

### 2.4. Statistical Analysis

All statistical analyses were conducted using SPSS 22.0 software (SPSS Inc. Chicago, IL, USA). Quantitative variables were expressed as the percentage or mean ± standard deviation. The Student’s *t*-test was applied for continuous variables, and the Chi-squared test was used for categorical data. Moreover, the normal distribution was determined for raw data using the Shapiro-Wilk test. To minimize the selection bias and possible confounding variables, PSM analysis was performed using logistic regression with the variables stated above. The PSM analysis was set at a ratio of 1:1 for BCLC stage C patients with sorafenib treatment and with or without previous experience of LR (LR versus non-LR). The survival analysis was assessed using the Kaplan–Meier method with a log-rank test. The impact factors associated with PFS and OS by univariate and multivariate analyses were analyzed using the Cox proportional hazards regression model. All *p*-values of less than 0.05 were considered statistically significant.

## 3. Results

### 3.1. Patient Characteristics

A total of 147 advanced HCC patients under sorafenib treatment were included in the cohort study. Before PSM, there were 84 patients (57.1%) aged 60 years and older. There were 120 male patients (81.6%) and 121 patients total (82.3%) with ECOG performance status 1. The etiology of liver disease included 43.5% hepatitis B virus (HBV) and 29.9% hepatitis C virus (HCV). The mean duration of sorafenib treatment was 5.9 ± 8.2 months. The prescribed daily dose of sorafenib was 400 mg and 800 mg for 105 and 42 patients, respectively. A total of 44 patients (29.9%) had undergone LR during the study period, and they had lower Child-Pugh class (*p* = 0.003) and higher percentage of the absence of ascites (*p* < 0.001) in comparison to the non-LR group (Table 1). After 1:1 PSM, 37 patients per group were recruited (in total, 74 patients), with was no significance in the selection of these patients (Table 1). The factors of a high tumor burden, defined as the presence of PVT, EHS, and tumor size > 3 cm or tumor nodules > 3, were found in 56, 54 and 37 patients, respectively.

### 3.2. Overall Survival and Progression-Free Survival Outcome

**Figure 2:**
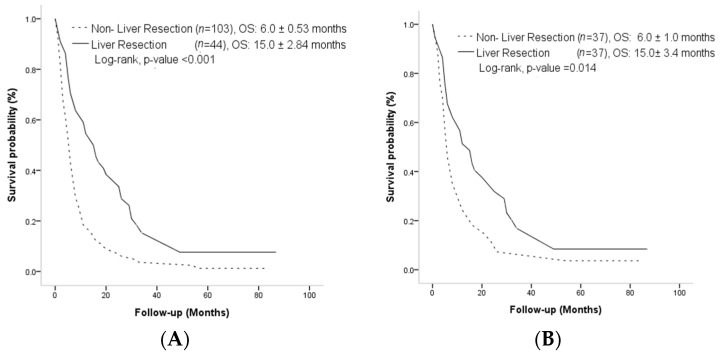
(**A**) Cumulative incidence of overall survival before propensity score matching. (**B**) Cumulative incidence of overall survival after propensity score matching.

**Figure 3:**
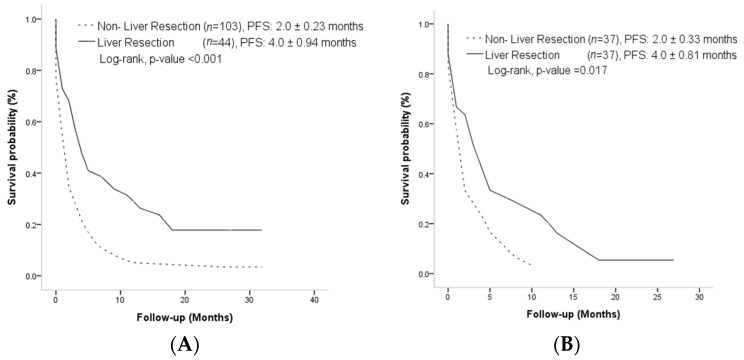
(**A**) Cumulative incidence of progression-free survival before propensity score matching. (**B**) Cumulative incidence of progression-free survival after propensity score matching.

There was statistical significance in the median OS of the LR and non-LR groups (15.0 ± 2.84 months vs. 6.0 ± 0.53 months, *p* < 0.001) (Figure 2A). After PSM, the median OS was still significantly longer in the LR group than the non-LR group (15.0 ± 3.4 months vs. 6.0 ± 1.00 months, *p* = 0.014) (Figure 2B). The median PFS was also significantly longer in the LR group than in the non-LR group, whether before PSM (4.0 ± 0.94 months vs. 2.0 ± 0.23 months, *p* < 0.001) (Figure 3A) or after PSM (4.0 ± 0.81 months vs. 2.0 ± 0.33 months, *p* = 0.017) (Figure 3B).

In the Cox regression model of univariate analysis, the experience of LR, an absence of ascites, a CTP score = 5, an AFP value lower than 400 ng/mL, and a tumor size > 3 cm or a number of tumor nodules ≥ 3 were all associated with better OS (*p* < 0.05) before PSM (Table 2). Only the experience of LR and the absence of ascites were associated with better PFS (*p* < 0.05) before PS matching (Table 3). After PSM, the only significant factor associated with longer OS (*p* = 0.02) and PFS (*p* = 0.004) was the experience of LR (Table 2 and Table 3).

In multivariate analyses, the experience of LR was the only significant factor with longer OS (*p* = 0.002) and PFS (*p* = 0.013) before PSM (Table 2 and Table 3). After PSM, the experience of LR was still significantly associated with both OS (*p* = 0.02) and PFS (*p* = 0.02) (Table 2 and Table 3).

## 4. Discussion

Sorafenib has been the standard treatment for BCLC stage C patients, based on the results of the SHARP and Asia Pacific trial in 2007 [13,14], and it has been approved by the Food and Drug Administration (FDA) for advanced HCC patients [16]. The updated AASLD practice guidelines still recommend the use of sorafenib for these patients if they are not appropriate candidates for any of the following treatments: atezolizumab and bevacizumab, tremelimumab and durvalumab, or durvalumab as monotherapy. Phase III trials testing nivolumab versus sorafenib failed to meet primary survival endpoints [17]. In the COSMIC 132 trial, the combination of cabozantinib and atezolizumab showed a significant benefit in the duration of progression-free survival, but the interim data did not show a significant survival benefit when compared to sorafenib as the first-line systemic treatment of patients with advanced HCC [18].

Another benefit of the use of sorafenib in advanced-stage HCC is the promising subsequent treatment plan if the sorafenib treatment fails. Patients with advanced HCC may benefit from regorafenib if they are tolerant to sorafenib [19]; from cabozantinib irrespective of their tolerance to sorafenib [20]; or from ramucirumab if their AFP level is >400 ng/mL [21]. Cabozantinib is also effective as a third-line treatment [20]. On the contrary, there have been no known agents that prolong the survival of HCC patients if all of the following treatments fail: atezolizumab and bevacizumab, tremelimumab and durvalumab, durvalumab, or lenvatinib. A lack of immune checkpoint inhibitor (ICI) cost-effectiveness may affect patients’ and medical physicians’ decisions around the treatment of advanced HCC [22,23]. These possible affecting factors may render patients more likely to choose an oral agent, like sorafenib, to take at home and save on travel costs, which is also likely to improve compliance. Sherrow et al. reported a preliminary result that first-line TKI followed by second-line immunotherapy is the most cost-effective strategy for advanced HCC [24]. In Taiwan, the cost of sorafenib has been covered by the National Health Insurance for advanced HCC patients since 2012, but the cost of ICIs is not covered.

In many clinical practice guidelines, LR is not recommended for intermediate and advanced HCC [15,25,26] because of the high tumor recurrence rate of 70% at 5 years after surgical resection [5,6,27]. The high risk of recurrence plays an important role in determining OS [28]. However, it is considered an acceptable treatment for patients in the Asian-Pacific area [29]. Many reports have shown that LR may prolong survival for intermediate- and advanced-stage HCC patients [8,9,10,11,30]. However, these reports did not focus on the management of postoperative recurrent advanced HCC with sorafenib. We intentionally enrolled BCLC stage B or C patients who had received surgical treatment because they had a higher risk of HCC recurrence. Recurrent or metastatic tumors behave differently from the original tumors, and it is possible that recurrent or metastatic tumors are more malignant because they were not eliminated by the initial therapy. Sorafenib has poor efficacy against intrahepatic metastases (derived from the primary tumor), as well as multicentric tumors arising spontaneously in the residual liver [31]. On the contrary, our study found that the administration of sorafenib for advanced HCC patients with previous experience of LR were conferred a longer OS and PFS, compared to those without previous LR, both before and after PSM (10 and 2 months longer). Furthermore, in multivariable Cox regression analysis, LR was associated with longer OS and PFS in advanced HCC patients who received sorafenib.

In 2015, Bruix et al. revealed that sorafenib as an adjuvant therapy did not provide a significant survival benefit for patients with early HCC after curative treatment in the STORM randomized trial [31]. However, Li et al. and Xia et al. demonstrated that adjuvant sorafenib treatment after hepatectomy for BCLC stage C HCC patients improved both PFS and OS [32,33]. Many retrospective studies from China have also reported that postoperative adjuvant sorafenib treatment improves survival outcomes for HCC patients with microvascular invasion, portal venous tumor thrombus, tumor rupture, or involvement of adjacent organs [34,35,36,37]. Nevertheless, choices for adjuvant therapy after curative hepatectomy for HCC remain an unmet medical need, and the ideal dose of adjuvant sorafenib was not confirmed by the studies described above. World-renowned clinical practice guidelines have also not incorporated sorafenib as an effective adjuvant treatment [15,25,26]. In the present study, we administered sorafenib as a “sequential therapy” after the development of postoperative recurrent advanced HCC, and we excluded patients using sorafenib as an adjuvant therapy. The advantage of sequential therapy over adjuvant therapy is to avoid unnecessary sorafenib for patients with a tumor-free status, as the side effects of sorafenib may impact quality of life and limit the treatment response [38].

The detailed mechanism of sorafenib in recurrent advanced HCC after surgical resection is not clear. Over-expression of VEGF in HCC patients is associated with a high recurrence rate and short postoperative survival [39], so the anti-VEGF effect of sorafenib may explain the survival benefit. The tumor burden has been considered to determine the prognosis for HCC after LR [40], possibly due to postoperative residual liver cell regeneration and the proliferation of residual trivial HCC cells. Sorafenib exhibits anti-angiogenesis and anti-tumor proliferation, resulting in tumor control [41]. Active and accelerated angiogenesis, along with its staging progression, may explain why early-stage HCC patients who had history of curative hepatectomy do not benefit from sorafenib, due to its low tumor recurrence rate and less aggressive angiogenesis after the operation. Some may argue that surgical patients have preserved liver function and are physically fit enough to undergo LR. However, the outcome of LR for intermediate and advanced HCC patients can be difficult to predict, and more clinical factors should be taken into account, such as possible insufficient reserved liver function, the possibility of incomplete tumor resection, and the high rate of recurrence [30]. In the cohort of sorafenib treatment in advanced HCC patients, the results of this study revealed that primary BCLC stage B or C HCC patients who had undergone LR and developed recurrent advanced HCC can still have better prognoses than those who did not undergo LR.

Short-interval follow-up is suggested for the early identification of recurrence after the liver operation [42], and early detection of advanced HCC may assist in the early administration of sorafenib. In Taiwan, the Health Promotion Administration declared in the Regulations for Cancer Care Quality Assurance Measures that HCC patients, after curative treatment, should be followed up with an imaging study in 2–3 months, and at least three times within the first year after treatment. Since LR is highly technical and risky, most liver surgeons request imaging studies and laboratory data even more frequently than this advisement, which may help with the early detection of recurrence.

Our study had several limitations. First, as a retrospective study, selection bias was an issue in the inclusion of patients and treatment options. The National Health Insurance program provides reimbursement for sorafenib with strict image and clinical criteria, which may lead to unintended bias. Although PSM and multivariable analysis were used in order to overcome this, an imbalance may have still existed between the LR and non-LR group. Second, this was an HCC case analysis from a single medical institute, thus the sample size was small, and lacking an external validation cohort. Finally, some prognostic factors, such as tumor differentiation, subsequent treatment, or other procedures in combination with the sorafenib treatment, were not documented due to a lack of detailed data.

## 5. Conclusions

In conclusion, our study demonstrated that LR confers an improved survival outcome in advanced HCC patients, compared with those who do not undergo LR. Patients with intermediate- or advanced-stage HCC should be encouraged to undergo LR if they are fit for the operation, as they may have a better prognosis even if they develop recurrent advanced HCC with sorafenib treatment.

## Figures and Tables

**Table 1:** Baseline characteristics and demographics of the patients before and after propensity score matching.

	Before Match (*n* = 147)			After Match (*n* = 74)		
Variables	LR Group (*n* = 44)	Non- LR Group (*n* = 103)	*p* Value	LR Group (*n* = 37)	Non-LR Group (*n* = 37)	*p* Value
Age			0.17			0.35
>60 years, *n* (%)	22 (50)	62 (60.3)		22 (59.5)	24 (64.9)	
Gender			0.26			0.24
Male, *n* (%)	35 (79.5)	85 (82.5)		29 (78.4)	31 (83.8)	
ECOG			0.4			0.24
PS 1, *n* (%)	35 (79.5)	86 (82.7)		29 (78.4)	31 (83.8)	
Etiology of liver disease			0.97			0.73
HBV, *n* (%)	21 (47.7)	43 (41.7)		16 (43.2)	16 (43.2)	
HCV, *n* (%)	14 (31.8)	30 (29.1)		13 (35.1)	11 (29.7)	
HBC + HCV, *n* (%)	3 (6.8)	4 (3.9)		2 (5.4)	3 (8.1)	
CTP score			0.003			1.00
6, *n* (%)	12 (23.7)	63 (60.6)		12 (32.4)	12 (32.4)	
Albumin			0.01			1.00
>3.5 g/L, *n* (%)	33 (75.0)	67 (65)		26 (70.3)	26 (70.3)	
Bilirubin			0.23			1.00
>2 mg/dL, *n* (%)	43 (97.7)	96 (93.2)		37 (100)	37 (100)	
Ascites			<0.001			1.00
Absent, *n* (%)	43 (97.7)	74 (71.8)		36 (97.3)	37 (100)	
AFP			0.88			0.66
<400 ng/mL, *n* (%)	28 (63.6)	53 (51.5)		22 (59.5)	25 (67.6)	
Tumor burden			<0.001			<0.001
PVT, *n* (%)	13 (29.5)	43 (41.7)		10(27.0)	20 (54.1)	
EHS, *n* (%)	28 (63.6)	26 (25.2)		24(64.9)	16 (29.7)	
Tumor size > 3 cm or number of tumor nodules > 3, *n* (%)	3 (6.8)	34 (33.0)		3(8.1)	6 (16.2)	

Statistics presented as the number (percent) and median ± standard deviation. Abbreviations: ECOG—Eastern Cooperative Oncology Group; HBV—Hepatitis B virus; HCV—Hepatitis C virus; CTP—Child-Turcotte-Pugh; INR—international normalized ratio; AFP—Alpha-Fetoprotein; PVT—portal vein thrombosis; EHS—extrahepatic spread.

**Table 2:** Univariable and multivariable Cox regression analysis for overall survival before and after propensity score matching.

Characteristics	Before Propensity Score Matching				After Propensity Score Matching			
	Univariable Analysis		Multivariable Analysis		Univariable Analysis		Multivariable Analysis	
	HR (95% CI)	*p* Value	HR (95% CI)	*p* Value	HR (95% CI)	*p* Value	HR (95% CI)	*p* Value
Treatment								
Non-LR LR	1 0.45 (0.30–0.66)	<0.001	1 0.48 (0.30–0.67)	0.002	1 0.55 (0.33–0.90)	0.02	1 0.48 (0.25–0.91)	0.02
Age								
>60 ≤60	1 0.98 (0.70–1.38)	0.93			1 0.92 (0.55–1.52)	0.75		
Gender								
Male Female	1 0.85 (0.54–1.34)	0.49			1 0.85 (0.45–1.60)	0.62		
ECOG								
PS 1 PS 0	1 0.58 (0.37–0.92)	0.21			1 0.55 (0.28–1.07)	0.08		
Etiology of hepatitis								
NBNC HBV HCV HBC + HCV	1 0.99 (0.63–1.55) 0.98 (0.61–1.57) 0.98 (0.41–2.39)	0.98 0.93 0.98			1 0.64 (0.20–2.07) 0.89 (0.31–2.56) 0.96 (0.33–2.79)	0.46 0.89 0.94		
CTP score								
6 5	1 0.62 (0.42–0.84)	0.003			1 0.76 (0.45–1.28)	0.31		
Albumin								
≤3.5 g/dL >3.5 g/dL	1 0.91 (0.64–1.30)	0.62			1 0.79 (0.43–1.22)	0.23		
Bilirubin								
≥2 mg/dL <2 mg/dL	1 0.83 (0.39–1.79)	0.64			1 0.48 (0.62–1.77)	0.46		
Ascites								
Presence Absence	1 0.48 (0.32–0.74)	<0.001	1 (Ref) 0.87 (0.45–1.68)	0.68	1 0.58 (0.14–2.42)	0.46		
AFP								
≥400 ng/mL <400 ng/mL	1 0.69 (0.49–0.97)	0.03	1 (Ref) 0.84 (0.57–1.23)	0.37	1 0.84 (0.51–1.40)	0.51		
Tumor burden								
PTV EHS Tumor size > 3 cm or number of tumor nodules ≥ 3	1 0.87 (0.56–1.56) 0.63 (0.40–0.99)	0.55 0.04	0.82 (0.51–1.31)	0.41	1 1.46 (0.66–3.21) 0.99 (0.45–2.19)	0.34 0.98		

Abbreviations: HR—hazard ratio; Ref—Reference; ECOG—Eastern Cooperative Oncology Group; PS—performance status; NBNC—Non-B, non-C; HBV—Hepatitis B Virus; HCV—Hepatitis C Virus; CTP—Child-Turcotte-Pugh; INR—international normalized ratio; AFP—alpha-fetoprotein; PVT—portal vein thrombosis; EHS—extrahepatic spread.

**Table 3:** Univariable and multivariable Cox regression analysis for progression-free survival before and after propensity score matching.

Characteristics	Before Propensity Score Matching				After Propensity Score Matching			
	Univariable Analysis		Multivariable Analysis		Univariable Analysis		Multivariable Analysis	
	HR (95% CI)	*p* Value	HR (95% CI)	*p* Value	HR (95% CI)	*p* Value	HR (95% CI)	*p* Value
Treatment								
Non-LR LR	1 0.51 (0.34–0.76)	0.001	1 0.55 (0.34–0.88)	0.013	1 0.45 (0.26–0.77)	0.004	1 0.43 (0.21–0.87)	0.02
Age								
>60 ≤60	1 0.97 (0.68–1.39)	0.89			1 0.58 (0.33–0.99)	0.83		
Gender								
Male Female	1 0.82 (0.52–1.31)	0.82			1 0.85 (0.45–1.60)	0.62		
ECOG								
PS 1 PS 0	1 0.79 (0.50–1.25)	0.32			1 0.55 (0.28–1.07)	0.08		
Etiology of hepatitis								
NBNC HBV HCV HBC + HCV	1 0.97 (0.61–1.54) 0.79 (0.48–1.30) 0.95 (0.39–2.33)	0.98 0.36 0.95			1 1.38 (0.76–2.85) 1.48 (0.73–3.13) 1.73 (0.55–5.44)	0.79 0.29 0.46		
CTP score								
6 5	1 0.79 (0.50–1.25)	0.32			1 1.35 (0.77–2.18)	0.31		
Albumin								
≤3.5 g/dL >3.5 g/dL	1 0. 80 (0.55–1.16)	0.25			1 1.37 (0.81–2.31)	0.23		
Bilirubin								
≥2 mg/dL <2 mg/dL	1 0.82 (0.38–1.77)	0.62			1 0.48 (0.62–1.77)	0.46		
Ascites								
Presence Absence	1 0.49 (0.31–0.76)	0.002	1 (Ref) 0.65 (0.32–1.31)	0.23	1 1.70 (0.41–7.03)	0.34		
AFP								
≥400 ng/mL <400 ng/mL	1 0.79 (0.56–1.13)	0.21						
Tumor burden								
PTV EHS Tumor size > 3 cm or number of tumor nodules ≥ 3	1 0.81 (0.54–1.21) 0.97 (0.61–1.53)	0.31 0.91			1 0.67 (0.40–1.15) 0.68 (0.31–1.50)	0.15 0.34		

Abbreviations: HR—hazard ratio; Ref—Reference; ECOG—Eastern Cooperative Oncology Group; PS—performance status; NBNC—Non-B, non-C; HBV—Hepatitis B Virus; HCV—Hepatitis C Virus; CTP—Child-Turcotte-Pugh; INR—international normalized ratio; AFP—alpha-fetoprotein; PVT—portal vein thrombosis; EHS—extrahepatic spread.

## Data Availability

The datasets generated for this study are available on request from the corresponding authors.
